# Apparentness of Unseen Rejection Episodes Post Lung rLTx Is Reliably Enabled by ddcfDNA Detection

**DOI:** 10.1111/tan.71005

**Published:** 2026-09-27

**Authors:** Aleksandra Gazikalovic, Christina Bade‐Döding, Murielle Verboom, Susann Zirzow, Michael Hallensleben, Rainer Blasczyk, Jens Gottlieb, Funmilola Josephine Haukamp

**Affiliations:** ^1^ Institute of Transfusion Medicine and Transplant Engineering, Hannover Medical School Hannover Germany; ^2^ Department of Respiratory Medicine and Infectious Diseases Hannover Medical School Hannover Germany

**Keywords:** allograft injury, cellular response, ddcfDNA, DSA, humoral response, liquid biopsy, lung transplantation, retransplantation

## Abstract

ddcfDNA emerged as a biomarker for post‐transplantation management strategies. Lung retransplant recipients represent a particularly vulnerable patient population with an increased risk of allograft injury. The demand for understanding if ddcfDNA levels differ between primary lung transplant recipients and lung retransplant recipients becomes obvious. Eight lung retransplant recipients and 50 primary lung transplant recipients with comparable HLA Class I and Class II mismatch scores were included in this retrospective study. During post‐transplant monitoring, ddcfDNA results were evaluated alongside data on patient humoral immune responses pre‐ and post‐transplantation. Antibody profiles of lung retransplant recipients did not differ significantly from those of their primary transplant comparisons. However, significantly higher ddcfDNA levels were observed in the retransplant cohort (median: 1.463%, IQR: 0.810–2.745) compared with primary transplant cohort (median: 0.550%, IQR: 0.338–0.935), *U* = 100.50, *Z* = −2.24, *p* = 0.012, *r* = 0.29. We identified that lung retransplant recipients exhibit increased allograft injury not solely attributable to humoral responses. Our study highlights that these patients face a higher immunological risk and supports the need for closer clinical follow‐up using a resilient biomarker such as ddcfDNA. Incorporating ddcfDNA as a surrogate marker into post‐transplant monitoring would provide early and unambiguous insights into allograft rejection, enabling prompt interventions to prevent unfavourable outcomes in this particularly fragile patient group.

## 1 Introduction

Lung diseases, alongside cardiovascular diseases, are among the leading causes of death in industrialised countries [1]. Due to the extensive polymorphism of HLA and restriction of available organs, HLA mismatches have to be accepted. Consequently, based on the immunogenicity of the respective mismatch, certain patients develop humoral immune responses against the transplanted organ. Chronic lung allograft dysfunction (CLAD) is the primary cause of a lower survival rate post‐lung transplantation (LTx) with a median survival rate of 6.2 years for adult transplant recipients and 4.8 years for recipients aged ≥ 60 [2]. Advancements have been made in surgical techniques, postoperative management and long term follow‐up, increasing the overall survival rate to 85% at 1‐year and 59% at 5‐years post‐Tx [3].

In severe cases of organ failure, solid organ retransplantation may become necessary. Indications for lung allograft retransplantation (rLTx) differ from those of primary LTx (pLTx) and most commonly include end‐stage CLAD, primary allograft failure and irreversible airway complications [4]. rLTx account for approximately 5% of annual lung transplants worldwide [4]. In most cases, retransplanted patients exhibit preformed anti‐HLA‐antibodies in the peripheral serum. Furthermore, anti‐HLA specific memory immune cells can be rapidly activated upon exposure to the second graft. Given the urgency for another organ along with the limited donor pool, some recipients will be re‐exposed to previously encountered mismatched HLA alleles, further elevating the risk of allograft rejection. Although short‐term outcomes post‐rLTx are comparable to outcomes of pLTx, long‐term survival remains poor, with retransplanted recipients experiencing more frequent hospitalisations and increased mortality beyond 1‐year post‐LTx [5]. In a large single‐centre study published recently, it has been reported that 1‐, 3‐, 5‐ and 10‐year survival rates following rLTx were 96%, 72%, 55% and 28%, respectively (median: 5.61 [3.30–8.51] years), whereas corresponding survival rates after pLTx were 88%, 74%, 63% and 39% (median: 7.34 [6.70–7.90] years) [6].

This understudied population of rLTx recipients lack sufficient research on the nature of immune responses to the second allograft, with mainly the patients' baseline characteristics, their functional status and the influence of surgical aspects of rLTx being described so far [4, 7, 8, 9]. Elucidating the mechanisms of immunological adverse reactions following rLTx would enable targeted therapies and decrease the likelihood of requiring a third transplant that may not be available in time.

Regular post‐LTx monitoring is crucial to detect acute rejection early and prevent CLAD. rLTx liquid biopsies represent a minimally invasive and paramount specific alternative to conventionally used methods—such as pulmonary function testing, imaging and transbronchial biopsies—enabling longitudinal monitoring of circulating biomarkers in peripheral blood, where elevated levels indicate allograft injury. To date, donor‐specific antibodies (DSAs) have been used routinely as liquid biomarkers of host versus graft responses. Yet, anti‐HLA DSAs are frequently undetectable post‐LTx, despite evidence of their significant impact on post‐LTx survival [10, 11, 12]. One limitation of using DSAs to monitor patient status is that they are typically detected only after approximately 3 months post‐LTx [13], meaning they cannot provide early insights into acute rejection. Moreover, the extensive endothelial surface area of the lung allograft enables adsorption of DSAs, thereby reducing their peripheral detectability—a phenomenon termed the ‘sponge effect’ [14, 15, 16]. Additionally, non‐HLA antibodies, such as antibodies against AT1R and ETAR, are associated with an increased risk of death post‐LTx, even if tested negative for anti‐HLA DSAs [17, 18]. Given the incomplete understanding of these antibodies' clinical relevance [19], their routine monitoring in solid organ transplantation has not been incorporated into standard practice. Hence, incomplete DSA data can result in insufficient appreciation of unacceptable HLA specificities, especially important in high‐risk patient cases, such as in rLTx patients.

Due to the mentioned drawbacks, relying solely on DSA detection for determination of lung allograft rejection is nowhere near enough. Although monitoring the emergence of DSAs post‐LTx can be beneficial, antibody determination provides only indirect evidence of immune activity and may not correlate with clinical rejection [20], and it has to be used in combination with other diagnostic methods; otherwise, underdiagnosis of subclinical antibody‐mediated rejection (AMR) could occur [21].

Unlike in kidney transplantation (KTx), where the incidence of DSA detection is higher (20% in LTx compared to 49% for KTx over 5‐years post‐Tx) [22, 23], LTx lacks a reliable biomarker of allograft injury. Thus, integrating additional liquid biopsy methods for post‐LTx follow‐up in the clinic would be highly beneficial. A promising biomarker for this purpose is donor‐derived cell‐free DNA (ddcfDNA). Cell‐free DNA (cfDNA) primarily originates from apoptosis, necrosis and active secretion. In a transplantation setting, in addition to the recipient's own cfDNA, ddcfDNA released by the allograft is also present in circulation. Published studies have reported that rising ddcfDNA levels following LTx strongly correlate with the occurrence of rejection [20, 24, 25], as well as with allograft failure and patient survival [26, 27, 28].

Although in KTx the detection of DSAs could imply presence of acute rejection episodes, in LTx ongoing graft injury may occur despite no detectable DSAs. In rLTx recipients, this limitation appears especially concerning. In this setting, subclinical acute rejection may remain undetected, potentially leading to repeated progressive and irreversible graft injury and, ultimately, life‐threatening outcomes if not recognised at an early stage. This observation aligns with evidence showing that kidney retransplant allografts exhibit significantly superior survival compared to rLTx allografts [8, 29, 30, 31].

Recognising lung allograft rejection at its earliest stages remains nearly impossible using conventional diagnostic methods because of their limitations mentioned [20, 26, 32]. In contrast, ddcfDNA has been reported to increase during these early stages, enabling the detection of lung allograft injury a median of 2.8 months prior to conventional monitoring methods such as spirometry or histopathological assessment [33], whereas preserving donor specificity even at a low acute rejection threshold [24, 34, 35, 36, 37]. In this context, an increase in ddcfDNA levels may signal the need for reassessment and adjustment of immunosuppressive therapy, supporting personalised treatment adjustments directed towards molecular manifestations of allograft injury [37, 38]. Therefore, improved non‐invasive post‐LTx surveillance strategy as this is urgently needed for this vulnerable cohort.

The present study aims to compare ddcfDNA levels between pLTx and rLTx recipients and to evaluate the potential role of ddcfDNA as a non‐invasive surrogate marker for post‐rLTx monitoring.

## 2 Methods and Materials

### 2.1 Patients and Data Collection

We included LTx recipients in whom an analysis of ddcfDNA was requested by the Department of Respiratory Medicine and Infectious Diseases, Hannover Medical School, between January 1, 2025 and November 30, 2025. No written consent has been obtained from the patients as there is no patient‐identifiable data included.

Inclusion criteria required HLA typing for both lung transplant recipients and donors at a minimum of five main loci (HLA‐A, ‐B, ‐C, ‐DRB1 and ‐DQB1), with pre‐transplant genomic DNA available from each for subsequent analysis. In addition, both ddcfDNA analysis and Luminex testing were conducted for every lung transplant recipient.

Eight patients had undergone rLTx. As a control group, we selected pLTx recipients with HLA Class I and II mismatch scores comparable to those of the lung rLTx recipients (*n* = 50) because both classes contribute to alloimmune recognition and the immunological impact of HLA disparities is not uniform across loci or epitopes [39]. HLA typing of recipients and lung donors, as well as antibody screening, were performed routinely by the Immunogenetics Laboratory at Institute for Transfusion Medicine and Transplant Engineering, Hannover Medical School. For all patients, organ donors were deceased (donation after brain death—DBD), and all transplantations were double lung transplantations. Cause of lung failure for first and/or second organ, ischemia time, immunosuppressive medication and induction therapy received were very diverse among all patients analysed.

### 2.2 Blood Collection and Monitoring Samples Preparation

Blood was collected in Cell‐free DNA BCT (STRECK, La Vista, USA) blood collection tubes at the Department of Respiratory Medicine and Infectious diseases, Hannover Medical School.

Blood plasma was isolated using double centrifugation. Blood samples were centrifuged at 1900 × g for 10 min in STRECK tubes, followed by 16000 × g for 10 min in 2 mL tubes. All plasma samples were stored at −80°C until further use. Extraction of cfDNA was performed using *QIAamp MinElute ccfDNA* kit (Qiagen, Hilden, Germany) following the manufacturer's instructions. Elution was done with 40 μL of ultra‐clean water. Quality of extracted cfDNA (purity and concentration of cfDNA) was examined by *TapeStation Cell‐free DNA ScreenTape assay* (Agilent, Santa Clara, USA) on *TapeStation* 4150 (Agilent).

### 2.3 Screening Samples Preparation

Genomic DNA (gDNA) was extracted from EDTA blood samples obtained from each patient prior to transplantation and from the corresponding donor. Extraction was done using *GenFind V3 Reagent Kit 384 preps* (Beckman Coulter, Brea, USA) with *Biomek i7* robot (Beckman Coulter) according to the manufacturer's instructions. gDNA concentration was determined using *Qubit 1X dsDNA Assay‐Kit* with High Sensitivity (HS) (Thermo Fisher Scientific, Waltham, USA) by measurement on *Qubit Flex Fluorometer* (Thermo Fisher Scientific) and diluted to 1 ng/μL with Dilution buffer (Devyser, Stockholm, Sweden) for library preparation.

### 2.4 Next Generation Sequencing (NGS)

Preparation of samples for sequencing was performed using the *One Lambda Devyser Accept cfDNA* kit (Devyser) according to the manufacturer's instructions. The protocol included two consecutive rounds of thermal cycling, PCR1 and PCR2, representing library generation and library indexing. PCR1 was used for amplifying 50 target short insertions and deletions (INDELs) in the genetic material. In PCR2, the amplified material was indexed—specific adapters containing unique index sequences were added to the amplicons. Due to the distinct sequence of each index, all samples could be differed when pooled in the final step and sequenced in the same NGS run.

Indexed libraries were pooled together in Screening and Monitoring pools that were afterwards purified using *Devyser Library Clean* kit (Devyser) according to the manufacturer's instructions. Purified libraries were then diluted until approximately 0.250 ng/μL.

The sequencing runs were done using *Illumina iSeq 100* (Illumina, San Diego, USA) with the *iSeq 100 i1 Reagents v2* (Illumina).

### 2.5 Sequencing Data Analysis

Sequencing output data was processed and analysed using *Advyser Solid Organs* software (Devyser) v. 1.2. Informative markers were determined in each pair consisting of lung transplant recipient and donor as described in Pettersson et al. [40]. Differentiation of recipient from donor (in case of rLTx, Donor 1 and Donor 2) cfDNA was done based on genetic sequence differences (INDELs) that vary between individuals, characterised as informative markers. By using a panel of 50 INDELs, it was possible to determine informative markers for each recipient and the respective organ donor. In rLTx cases, informative markers were determined for both organ donors separately and used in ddcfDNA analysis. The proportion of ddcfDNA was quantified and expressed as percentage of ddcfDNA within the total cfDNA pool for each sample.

### 2.6 Antibody Screening

DSAs from peripheral serum were determined using *LIFECODES LSA Class I* and *LIFECODES LSA Class II* kits (Werfen, Barcelona, Spain) with Luminex *FLEXMAP 3D* (Luminex Corporation, Austin, USA). The results were analysed using the *xPONENT* software (Luminex Corporation). For the specification of the HLA‐antibodies, the *hlaSYSTEM* (AVALAS, Isernhagen, Germany) was used.

### 2.7 Statistical Analysis

ddcfDNA values detected in rLTx recipients were compared to those in pLTx values using the Mann–Whitney *U* test, complemented by the calculation of the effect size *r*. This nonparametric test was chosen because the data were not normally distributed and the group sizes were unequal. A *p* value < 0.05 was considered statistically significant. Antibody detection (pre‐LTx, post‐LTx, post‐LTx DSA) was compared between rLTx and pLTx recipients using a Fisher's exact test, and effect sizes were expressed as odds ratios (OR) with 95% confidence intervals (CI). A *p* value < 0.05 was considered statistically significant. Age distribution was assessed using an unpaired *t*‐test. Time from LTx to ddcfDNA measurement was assessed using Mann–Whitney *U* test. The association between age and ddcfDNA levels was assessed using Spearman's rank correlation. Analysis was performed separately for each group to account for potential group‐specific differences. This nonparametric test was chosen because the data were not normally distributed and the group sizes were unequal. Spearman *r* and corresponding two‐tailed *p* values were calculated with statistical significance defined as *p* < 0.05. All analyses were performed using *GraphPad Prism* version 8.2.0 for Windows (GraphPad Software, La Jolla, USA).

## 3 Results

We analysed the presence of anti‐HLA antibodies pre‐ and post‐LTx and evaluated ddcfDNA levels in eight rLTx recipients and 50 pLTx recipients with comparable HLA Class I and Class II mismatch scores to account for the hierarchical contribution of HLA disparities to alloimmune risk. The median number of HLA Class I mismatches was 5 (IQR: 3.5–6) in rLTx recipients compared with 5 (IQR: 5–6) in pLTx recipients (*p* = 0.683). The median number of HLA Class II mismatches was 4 (IQR: 3.25–4.75) and 4 (IQR: 4–5), respectively (*p* = 0.448) (Table S1).

Among the 50 pLTx recipients, 35 (70%) were male and 15 (30%) were female, whereas only one (12.5%) recipient in the rLTx group was male and seven (87.5%) were female. The median age at LTx in the pLTx cohort was 56 (IQR: 49.5–61) years. In the rLTx cohort, recipients underwent their pLTx at a median age of 20 (IQR: 16.75–32) years and their rLTx at a median age of 28.5 (IQR: 22–43.25) years. The median interval between pLTx and rLTx was 4 (IQR: 2–11) years (Table 1). In our study cohort, primary disease leading to a need for transplantation, induction therapy and immunosuppressive medication received were too heterogeneous to allow a comprehensive analysis of patient overlap. Time from LTx to ddcfDNA measurement varied among both pLTx patients and rLTx patients, as this was a retrospective observational study without predefined sampling timepoints. Timing of sampling between pLTx and rLTx differed, with median time from rLTx to ddcfDNA measurement being 520.5 days (IQR: 43.25–2695), whereas for the pLTx it was 664 days (IQR: 130–3036). This difference did not reach statistical significance (*p* = 0.5570).

**TABLE 1:** Baseline patient characteristics.

	pLTx cohort (*n* = 50)	rLTx cohort (*n* = 8)
Gender		
Male	35 (70%)	1 (12.5%)
Female	15 (30%)	7 (87.5%)
Age at pLTx (years)	56 (IQR: 49.5–61)	20 (IQR: 16.75–32)
Age at rLTx (years)		28.5 (IQR: 22–43.25)
Interval between pLTx and rLTx (years)		4 (IQR: 2–11)

*Note:* Data are presented as *n* (%) or median (IQR).

Abbreviations: IQR, interquartile range; pLTx, primary lung transplantation; rLTx, repeated lung transplantation.

Informative markers were detected in ddcfDNA from all screened patients for both Donor 1 and Donor 2. The number of infomative markers per donor ranged from two to seven. Therefore, it was possible to distinguish and quantify origin of ddcfDNA in all cases. Informative markers for each rLTx patient, Donor 1 and Donor 2, along with the corresponding ddcfDNA measurements, are given in Table S2.

Anti‐HLA antibodies were detected before LTx in 30% (*n* = 15) of pLTx recipients and 63% (*n* = 5) of rLTx recipients. Particularly for rLTx recipients, there was a slight predominance of antibodies against HLA Class II. Post‐LTx, anti‐HLA antibodies were detected in 48% (*n* = 24) of pLTx recipients, 63% (*n* = 15) of these were DSAs. In comparison, anti‐HLA antibodies were detected in 75% (*n* = 6) of rLTx recipients and solely 33% (*n* = 2) of these were DSAs. Again, the detected antibodies and DSAs were primarily directed against HLA Class II molecules: 40% (*n* = 20) of pLTx recipients developed anti‐HLA Class II antibodies, and 22.0% (*n* = 11) developed anti‐HLA Class II DSAs post‐LTx. In addition, 50% (*n* = 4) of rLTx recipients developed anti‐HLA Class II antibodies and 25% (*n* = 2) anti‐HLA Class II DSAs post‐rLTx. However, no statistically significant differences were observed when comparing the presence of anti‐HLA antibodies pre‐ and post‐(r)LTx in pLTx and rLTx recipients (Table 2).

**TABLE 2:** Pre‐ and post‐LTx anti‐HLA antibody and DSA profiles.

	pLTx cohort (*n* = 50)	rLTx cohort (*n* = 8)	OR (95% CI)	*p*
Pre‐LTx Ab^+^	15 (30%)	5 (63%)	3.9	0.110
Anti‐HLA Class I	9 (18%)	2 (25%)	[0.8, 15.8]	
Anti‐HLA Class II	11 (22%)	3 (38%)		
Post‐(r)LTx Ab^+^	24 (48%)	6 (75%)	3.3	0.256
Anti‐HLA Class I	11 (22%)	3 (38%)	[0.7, 16.7]	
Anti‐HLA Class II	20 (40%)	4 (50%)		
Post‐(r)LTx DSA^+^	15 (30%)	2 (25%)	1.0	> 0.999
Anti‐HLA Class I	5 (10%)	0 (0%)	[0.2, 4.8]	
Anti‐HLA Class II	11 (22%)	2 (25%)		
% DSA^+^ of post‐(r)LTx Ab^+^	63%	33%		

*Note:* Data are presented as *n* (%).

Abbreviations: Ab, antibodies; CI, confidence interval; DSA, donor‐specific antibodies; LTx, lung transplantation; OR, odds ratio.

In contrast, a Mann–Whitney *U* test indicated significantly higher ddcfDNA values in rLTx recipients (median: 1.463%, IQR: 0.810–2.745) compared with pLTx recipients (median: 0.550%, IQR: 0.338–0.935), *U* = 100.50, *Z* = −2.24, *p* = 0.012, *r* = 0.29 (Figure 1). The results reflect differences in the distributions, with small‐to‐medium effect size.

**FIGURE 1:**
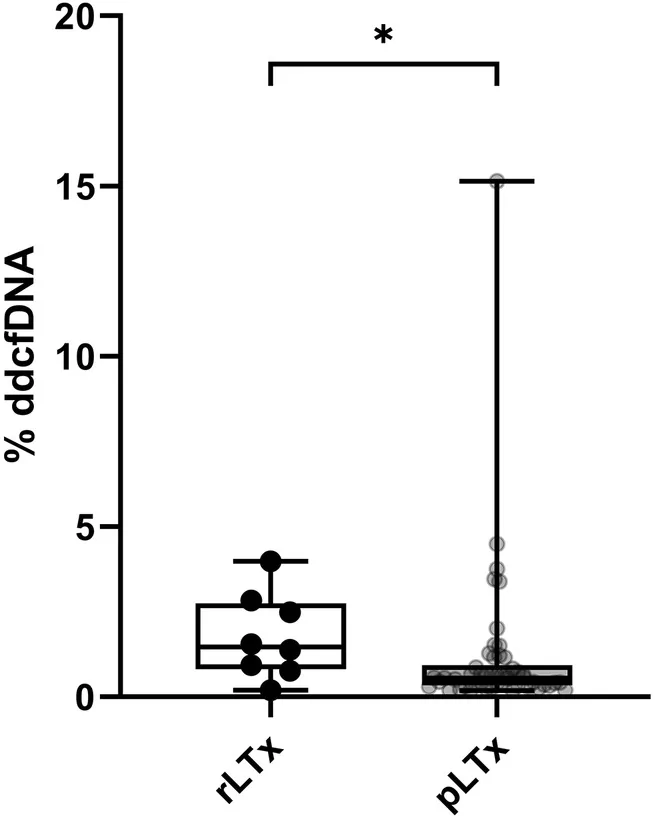
ddcfDNA values are significantly higher in rLTx recipients when compared to pLTx recipients. Boxplot showing the distribution of values in rLTx recipients (*n* = 8) and pLTx recipients (*n* = 50). Box represents the interquartile range (25th–75th percentile), the horizontal line indicates the median, and whiskers show minimum and maximum values. Individual data points are overlaid. Statistical comparison was performed using the Mann–Whitney U test (*U* = 100.50, *Z* = −2.24, *p* = 0.012, *r* = 0.29); *p < 0.05.

Since rLTx recipients are much younger in age than pLTx recipients, we furthermore compared ddcfDNA levels in age‐matched rLTx and pLTx (28–55 years) recipients with respect to the age at time of sample collection. An unpaired *t*‐test verified that with respect to age, both groups did not differ significantly (*p* = 0.5156) (Figure 2A). Furthermore, a significant difference in ddcfDNA values could not be detected between rLTx recipients (median: 1.040%, IQR: 0.560–3.235) and age‐matched pLTx recipients (median: 0.560, IQR: 0.430–0.730), *U* = 21, *Z* = −1.44, *p* = 0.084, *r* = −0.32 (Figure 2B). However, differences in distribution with a small‐to‐medium effect size could be observed implying a potential trend towards lower ddcfDNA values in pLTx recipients.

**FIGURE 2:**
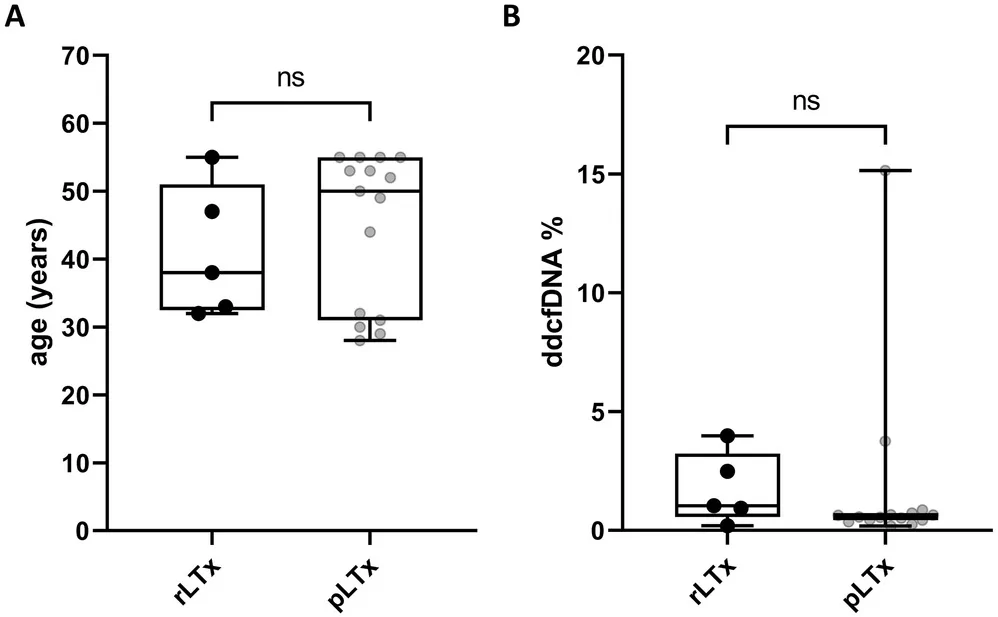
ddcfDNA level comparison in age‐matched rLTx and pLTx recipients (28–55 years). (A) Unpaired *t*‐test to verify that both groups do not differ in age. (B) Comparison of ddcfDNA values in age‐matched rLTx and pLTx recipients. Boxplot showing the distribution of values in rLTx recipients (*n* = 5) and pLTx recipients (*n* = 15). Box represents the interquartile range (25th–75th percentile), the horizontal line indicated the median, whiskers show minimum and maximum values. Individual data points are overlaid. Statistical comparison of ddcfDNA levels was performed using the Mann–Whitney U test (*U* = 21, *Z* = −1.44, *p* = 0.084, *r* = −0.32).

Furthermore, we analysed whether there is a correlation between ddcfDNA levels and age for age‐matched rLTx and pLTx recipients as well as for the whole cohorts using Spearman's rank correlation (Table 3). In the age‐matched subgroups, a significant negative correlation between age and ddcfDNA levels was initially observed in pLTx recipients (Spearman *r* = −0.56, *p* = 0.033), whereas no significant association was detected in rLTx recipients (Spearman *r* = −0.50, *p* = 0.45). However, these findings were not confirmed in the whole pLTx recipient analysis (Spearman *r* = −0.16, *p* = 0.27).

**TABLE 3:** Correlation between age and ddcfDNA levels.

	*n*	Age range (years)	Spearman's *r*	*p*
Age‐matched rLTx cohort	5	32–55	−0.500	0.450
Whole rLTx cohort	8	22–55	−0.159	0.270
Age‐matched pLTx cohort	15	28–55	−0.557	0.033
Whole pLTx cohort	50	28–71	−0.180	0.672

## 4 Discussion

In this single‐centre retrospective study, we compared the presence of anti‐HLA antibodies pre‐ and post‐LTx and ddcfDNA levels between rLTx recipients and pLTx recipients. The recipients were selected according to their HLA Class I and Class II mismatch scores. Mismatch scores that are calculated based on donor to recipient direction are a first virtual measurement of histocompatibility and are not based on available recipient anti‐HLA Abs. HLA Class I and Class II mismatch scores both contribute to alloimmune recognition; the immunological impact of HLA disparities is not uniform across loci or epitopes. Particularly, Class II mismatches are often considered more strongly associated with DSA development and clinically relevant humoral alloimmunity [41]. Recipients of a pLTx were predominantly male (70%), whereas the majority of rLTx recipients were female (87.5%). A reasonable explanation could be that prior sensitisation events, particularly pregnancies, increase the risk of antibody‐mediated transplant rejection [42, 43]. Furthermore, pLTx recipients are much more advanced in age than rLTx recipients. The median age of pLTx recipients in our study is consistent with the current report of the International Thoracic Organ Transplant (TTX) Registry of the International Society of Heart and Lung Transplantation that indicates a median recipient age of 58 years [44]. rLTx recipients are significantly younger, probably because recipient selection for rLTx is performed exceedingly carefully, and risk factors such as older age, need for early rLTx within 5 years, and female sex need to be considered [5]. The interval between pLTx and rLTx in our study was 4 (IQR: 2–11) years, which slightly differs from a reported median graft survival of 6.2 years [8]. Overall, rLTx patients had a shorter interval post‐transplantation to blood sample collection for ddcfDNA testing. However, Mann–Whitney *U* test did not reveal a statistically significant difference (*p* = 0.5570), and therefore this shorter interval is unlikely to have an influence on measured ddcfDNA values. Shorter post‐transplant intervals to sample collection in these patients could reflect stricter routine monitoring in rLTx patients (indication for routine biopsy) or sampling triggered by clinical symptoms (indication for for‐cause biopsy). In rLTx patients, ddcfDNA was quantified from both Donor 1 and Donor 2. Measured Donor 1 values were low, below the limit of detection (0.1%) [40], consistent with the fact that the first lungs had been explanted from the patients. Accordingly, little (originating from residual tissue) or no signal at all was detectable.

Our findings are coherent with a previous report on ddcfDNA monitoring in multiple kidney transplantation settings, where comparable numbers of informative markers were detected and ddcfDNA originating from different donor kidneys could be quantified. Similar to our results, this NGS‐based ddcfDNA analysis showed low or no ddcfDNA from explanted kidneys, proving that accurate determination of ddcfDNA origin is realistically achievable [45].

When comparing rLTx and pLTx recipients, the proportion of recipients with pre‐LTx anti‐HLA antibodies (63% in rLTx vs. 30% in pLTx recipients) and post‐LTx anti‐HLA antibodies (75% vs. 48%) suggests a potential difference. The analysis of anti‐HLA antibodies pre‐LTx revealed an OR of 3.9 (95% CI [0.8, 15.8]) indicating a higher sensitisation in rLTx recipients, however the result was not statistically significant (*p* = 0.110). The OR for post‐LTx anti‐HLA antibody detection was 3.2 (95% CI [0.7, 16.7]) also indicating a trend towards higher odds for anti‐HLA antibody development in rLTx recipients (*p* = 0.256). Interestingly, the proportion of DSAs among anti‐HLA antibodies detected post‐LTx was lower for rLTx recipients (33%) than for pLTx recipients (63%). However, statistical analysis revealed an OR of 1.0 (95% CI [0.2, 4.8], *p* = > 0.999) suggesting no significant difference between the two groups. In both groups, post‐LTx anti‐HLA antibodies and DSAs were particularly directed against HLA Class II molecules. Interestingly, DSAs were most frequently directed against HLA‐DQ, occurring in 67% of pLTx recipients as well as in both rLTx recipients with detected DSAs (data not shown). This data align with other studies also showing a predominance of de novo anti‐HLA‐DQ DSAs post‐LTx [46, 47]. Taken together, the wide CIs and the lack of statistical significance suggest no evidence of a difference in anti‐HLA antibody and DSA profiles between rLTx and pLTx recipients.

In contrast, we found that rLTx was associated with significantly higher ddcfDNA levels (*U* = 100.50, *Z* = −2.25, *p* = 0.012, *r* = 0.29), despite similar HLA Class I and II mismatch scores, and anti‐HLA antibody and DSA profiles. Although HLA mismatch is a key determinant of alloimmune activation, it does not fully capture the complexity of sensitisation and graft injury, particularly in patients with prior allograft exposure. Importantly, ddcfDNA is increasingly recognised as a sensitive marker of allograft injury in LTx, with superior performance characteristics compared with traditional markers such as spirometry or DSAs alone [24, 33, 34, 35, 36, 37].

Elevated ddcfDNA levels (> 1%) are found to be associated with rLTx patients, yet they were detected in some pLTx patients as well. A rise in ddcfDNA levels indicates allograft damage, whether the cause is immune‐mediated or due to another type of physical injury to the organ. Although elevated ddcfDNA suggests an alloimmune response resulting in tissue damage and subsequent release of donor DNA from the affected cells, other explanations for its elevation could be presence of an underlying respiratory infection or mechanical injury of the lung, especially if measured in the early post‐transplantation period (cold ischemic time, reperfusion injury, surgical trauma). Therefore, it could be assumed that elevated ddcfDNA values in some pLTx patients stem from other type of organ injury, and not a robust alloimmune response.

Comparing ddcfDNA levels between age‐matched rLTx and pLTx recipients using Mann–Whitney *U* test did not reveal a statistically significant difference (*U* = 21, *Z* = −1.44, *p* = 0.084, *r* = −0.32), although a small‐to‐medium effect size was observed which suggests a potential tendency towards lower ddcfDNA levels in pLTx recipients. However, small sample sizes could explain the lack of statistical significance. In summary, no clear conclusion can be drawn. When assessing the relationship between age and ddcfDNA levels using Spearman's rank correlation, we initially observed a potential negative correlation in pLTx recipients (Spearman *r* = −0.56, *p* = 0.033) suggesting that higher ddcfDNA values were detected in younger recipients, however, this finding was not reproducible in the whole pLTx cohort (Spearman *r* = −0.16, *p* = 0.27). This discrepancy is most likely explained by reduced statistical power in the analysis of age‐matched cohorts due to small group sizes. Additionally, we could not detect a significant correlation between age and ddcfDNA levels in the age‐matched or whole rLTx cohort. Thus, the data do not support age as a clinically relevant predictor of ddcfDNA levels in our cohorts.

To our knowledge, this is the first study to directly investigate ddcfDNA dynamics in rLTx, a population that remains underrepresented in biomarker research due to its rarity. Our findings support its potential as an adjunctive tool in more complex transplant populations, including rLTx recipients, where early detection of graft injury is particularly valuable.

Although our sample size of rLTx recipients was modest (*n* = 8), it reflects the relative rarity of rLTx that accounts for approximately 5% of annual LTx [4]. The consistency of the signal despite small numbers suggests that the observed differences are meaningful.

A limitation of this study is that ddcfDNA was measured solely as fractional abundance, which may be influenced by variation in total recipient cfDNA and therefore may not fully reflect the absolute burden of ddcfDNA. Aditionally, as a retrospective observational study, systematic longitudinal monitoring of ddcfDNA was not performed. Measurements were taken only at timepoints of clinically indicated visits, resulting in irregular sampling, with some patients evaluated in the early post‐transplant period and others only at later stages. Consequently, the expected post‐transplant decline in ddcfDNA values could not be captured, and ddcfDNA temporal dynamics could not be reliably compared between pLTx and rLTx.

In summary, our findings indicate that rLTx recipients exhibit higher ddcfDNA levels than pLTx recipients, despite no significant differences in the occurrence of antibodies and/or DSAs post‐LTx. This suggests that immunological mechanisms beyond humoral pathways, as well as non‐immunological factors unique to rLTx, may contribute to elevated ddcfDNA release.

## Author Contributions

Conceptualisation: A.G., C.B.‐D. and F.J.H. Methodology: A.G., C.B.‐D. and M.V. Software: M.H. and F.J.H. Validation: A.G., M.V. and F.J.H. Formal analysis: A.G., C.B.‐D., M.V. and F.J.H. Investigation: A.G., C.B.‐D. and F.J.H. Resources: R.B. and J.G. Writing – original draft: A.G., S.Z. and F.J.H. Writing – review and editing: C.B.‐D., M.V., M.H., R.B. and J.G. Supervision: C.B.‐D. and F.J.H. All authors have read and agreed to the published version of the manuscript.

## Ethics Statement

The study was approved by the Ethics Committee of Hannover Medical School (Nr. 11511_BO_K_2024, 14 November 2024).

## Conflicts of Interest

The authors declare no conflicts of interest.

## Supporting information


**Table S1:** Comparison of HLA Class I and Class II mismatch scores between rLTx and pLTx recipients.
**Table S2:** Informative markers for rLTx patients, Donor 1 and Donor 2 and corresponding ddcfDNA measurements.

## Data Availability

Research data are not shared.
