# Supplementary material for: Apparentness of Unseen Rejection Episodes Post Lung rLTx Is Reliably Enabled by ddcfDNA Detection
Source: HLA. 2026 Sep 27;108(4):e71005. doi: 10.1111/tan.71005 (PMC13617177; doi:10.1111/tan.71005)
Supplement: Supplementary file 1 [file TAN-108-e71005-s001.docx]

**Supplementary Table 1:** Comparison of HLA class I and class II mismatch scores between rLTx and pLTx recipients.

|  | rLTx  *(n = 8)* | pLTx  *(n = 50)* | *p*-value |
| --- | --- | --- | --- |
| HLA class I mismatches | 5 (IQR: 3.5—6) | 5 (IQR: 5—6) | 0.683 |
| HLA class II mismatches | 4 (IQR: 3.25—4.75) | 4 (IQR: 4—5) | 0.448 |

**Supplementary Table 2:** Informative markers for rLTx patients, donor 1 and donor 2 and corresponding ddcfDNA measurements

| rLTx  patient | Informative markers | | | Measurement 1  (ddcfDNA %) | | Measurement 2  (ddcfDNA %) | |
| --- | --- | --- | --- | --- | --- | --- | --- |
|  | **Recipient** | **Donor 1** | **Donor 2** | **Donor 1** | **Donor 2** | **Donor 1** | **Donor 2** |
|  |  |  |  |  |  |  |  |
| 1 | 8 | 2 | 2 | 0.01 | 1.04 | 0.00 | 1.72 |
| 2 | 7 | 4 | 6 | 0.20 | 1.91 | 0.00 | 1.18 |
| 3 | 2 | 3 | 7 | 0,00 | 0,19 |  |  |
| 4 | 2 | 4 | 5 | 0,00 | 0,93 |  |  |
| 5 | 4 | 7 | 5 | 0,00 | 2,83 |  |  |
| 6 | 3 | 5 | 7 | 0,01 | 3,98 |  |  |
| 7 | 4 | 4 | 6 | 0,02 | 0,77 |  |  |
| 8 | 3 | 3 | 4 | 0,05 | 2,49 |  |  |
